# T Cell Activation Inhibitors Reduce CD8+ T Cell and Pro-Inflammatory Macrophage Accumulation in Adipose Tissue of Obese Mice

**DOI:** 10.1371/journal.pone.0067709

**Published:** 2013-07-02

**Authors:** Vince N. Montes, Michael S. Turner, Savitha Subramanian, Yilei Ding, Martha Hayden-Ledbetter, Sonya Slater, Leela Goodspeed, Shari Wang, Mohamed Omer, Laura J. Den Hartigh, Michelle M. Averill, Kevin D. O’Brien, Jeffrey Ledbetter, Alan Chait

**Affiliations:** 1 Division of Metabolism, Endocrinology and Nutrition, University of Washington, Seattle, Washington, United States of America; 2 Benaroya Research Institute, Seattle, Washington, United States of America; 3 Division of Rheumatology, University of Washington, Seattle, Washington, United States of America; 4 Division of Cardiology, University of Washington, Seattle, Washington, United States of America; University of Padova, Italy

## Abstract

Adipose tissue inflammation and specifically, pro-inflammatory macrophages are believed to contribute to insulin resistance (IR) in obesity in humans and animal models. Recent studies have invoked T cells in the recruitment of pro-inflammatory macrophages and the development of IR. To test the role of the T cell response in adipose tissue of mice fed an obesogenic diet, we used two agents (CTLA-4 Ig and anti-CD40L antibody) that block co-stimulation, which is essential for full T cell activation. C57BL/6 mice were fed an obesogenic diet for 16 weeks, and concomitantly either treated with CTLA-4 Ig, anti-CD40L antibody or an IgG control (300 µg/week). The treatments altered the immune cell composition of adipose tissue in obese mice. Treated mice demonstrated a marked reduction in pro-inflammatory adipose tissue macrophages and activated CD8+ T cells. Mice treated with anti-CD40L exhibited reduced weight gain, which was accompanied by a trend toward improved IR. CTLA-4 Ig treatment, however, was not associated with improved IR. These data suggest that the presence of pro-inflammatory T cells and macrophages can be altered with co-stimulatory inhibitors, but may not be a significant contributor to the whole body IR phenotype.

## Introduction

Obesity is associated with significant comorbidity, including increased risk for type 2 diabetes and cardiovascular disease [1]. It is believed that the chronic low grade inflammation that accompanies obesity contributes to systemic insulin resistance, which is a component of type 2 diabetes [2]. In addition, it is widely accepted that inflammation plays a key role in the development of atherosclerosis [3]. Obesity is characterized by the accumulation of diverse immune cell types in adipose tissue [4]. Recruitment of pro-inflammatory macrophages to adipose tissue appears to be a cardinal feature of obesity [5]. Characterization of these cells by cell membrane markers includes those that are positive for F4/80, CD11b and CD11c. It was also determined that pro-inflammatory cytokines produced by these macrophages, such as TNF-α, can interfere with insulin signaling [6].

In addition to macrophages, T lymphocytes of the adaptive immune response are recruited to obese adipose tissue. Accumulation of CD8+ T cells appears to precede the appearance of pro-inflammatory macrophages [7]. Moreover, the ratio of Th1 to Th2 subsets of CD4+ T cells is increased in obesity [8], suggesting a role for pro-inflammatory Th1 cells. Also, anti-inflammatory T regulatory cells (Treg) are reduced in adipose tissue from obese mice [9]. Thus, T cells appear to play an important role in obesity-associated inflammation.

T cells typically respond to antigens presented by MHC molecules. MHC class I antigens are presented by most nucleated cells activate cytotoxic CD8+ T cells. MHC class II antigen presentation is restricted to professional antigen presenting cells (APC), and activates CD4+ T cells, which can be helper or regulatory T cells. Antigen presented to a T cell binds to its T cell receptor leading to the first step of activation. Primed T cells then receive a second signal, termed co-stimulation, from CD80/86 on the antigen presenting cells, which binds to CD28 on the T cell membrane. This two-step process leads to full activation of most T cells. This is a simplistic representation of T cell activation and other co-stimulatory and co-inhibitory pathways also exist [10]. Another major pathway for T cell co-stimulation is the CD40-CD40L pathway. CD40 ligand (CD40L or CD154) on T cells binds to CD40 on APC to act as an indirect, but synergistic co-stimulatory pathway. The ligation of CD40 augments the antigen presenting function of multiple APC such as macrophages, dendritic cells and B cells. Although we present a simplistic view of CD40 ligation as a T cell response pathway, it is very likely that blocking this pathway affects multiple actions within the inflammatory cascade. This pathway can be inhibited by an anti-CD40L antibody. The CD80/86-CD28 pathway can be inhibited by a molecule called abatacept, which is currently FDA approved to treat rheumatoid arthritis. Inhibition of these pathways is a viable therapeutic option for ameliorating diseases that are associated with T cell activation.

Due to the presence and proposed roles of T cells in adipose tissue, we hypothesized that reducing T cell activation with co-stimulatory inhibitors would reduce pro-inflammatory T cell and macrophage accumulation in adipose tissue and concomitant insulin resistance. To test this hypothesis, mice were treated with the murine versions of CTLA-4 Ig and anti-CD40L antibody, while being maintained on an obesogenic and pro-inflammatory diet.

## Methods

### Reagents

The mCTLA4-mIgG2a cell line was generated in the laboratory of Jeffrey Ledbetter, with approval of the University of Washington Institutional Animal Care and Use Committee (Protocol Number: 4228-01). Stable transfectants were generated by high copy electroporation of CHO DG44 cells (obtained from L. Chasin, Columbia University) as described previously [11]. Anti-CD40L antibody was from the MR1 clone, a hamster hybridoma producing mAb to murine gp39 (CD40L) provided by Randy Noelle and is available from American Type Culture Collection (Catalog # CRL-2580, ATCC, Manassas, VA). IgG was 60.3, a murine IgG2a non-reactive with murine antigens [12]. To generate antibody or fusion proteins for *in vivo* assays, culture supernatants were collected by centrifugation from spent hybridoma cultures (MR1) or spent CHO cultures expressing the CTLA4Ig fusion protein. Supernatants were diluted 1∶1 with protein G binding buffer (Catalog # PI-21011, ThermoFisher Scientific, Waltham, MA) filtered through 0.2 µm filters and passed over Protein G-agarose (Catalog # PI-22852, ThermoFisher Scientific, Dallas, TX). The column was conditioned with Protein G binding buffer, then loaded by gravity flow to allow binding of fusion protein, and washed with column wash buffer (protein G binding buffer) prior to elution. Bound protein was eluted using IgG elution buffer (Catalog #PI-21004, ThermoFisher Scientific) and dialyzed against D-PBS (Hyclone, ThermoFisher Scientific) pH 7.4. After dialysis, protein was filtered using 0.1 µM filter units, and aliquots tested for endotoxin contamination using Pyrotell LAL gel clot system single test vials (Catalog # G2006, Associates of Cape Cod, East Falmouth, MA).

### Mice

Male C57BL/6 mice at 8 weeks of age were purchased from Jackson Laboratories (Sacramento, CA, USA). Mice were fed either a chow diet or a high fat, high sucrose-containing diet with added cholesterol (HFHSC or obesogenic diet w/cholesterol 0.15%; BioServ No.F4997, Frenchtown, NJ) for 16 weeks. Four groups of mice (n = 12 per group) were fed the HFHSC diet and either received intraperitoneal IgG injection (as a protein injection control), CTLA-4 Ig, anti-CD40L antibody (150 µg twice weekly) or no injection. A fifth group of mice received a chow diet as a negative control. Each group received a similar volume of injection. Animals were housed in a specific pathogen-free vivarium and maintained on a 12-hour light/dark cycle in a temperature-controlled room. Animals were weighed weekly. Food intake was measured at 11 weeks. At the end of the study, animals were sacrificed by inhalation anesthesia and exsanguination and tissues (liver and epididymal adipose tissues) were harvested, weighed, snap-frozen and stored at −80°C until further analysis. Body composition was assessed non-invasively at 12 weeks in unanesthetized mice using NMR spectrometry in the University of Washington’s Nutrition Obesity Research Center’s (NORC) Energy Balance and Glucose Metabolism Core Laboratory. This study was carried out in strict accordance with the recommendations in the Guide for the Care and Use of Laboratory Animals of the National Institutes of Health. Animal protocols were approved by the University of Washington Institutional Animal Care and Use Committee (Protocol Number: 3104-1). All efforts were made to minimize suffering.

### Blood Chemistry

Blood (50 µL) was collected after a 4 hour fast from the retro-orbital sinus every 4 weeks until sacrifice. Plasma glucose, triglycerides and cholesterol were measured using colorimetric assays, and plasma insulin was measured using a commercially available kit, as described previously [13]. Plasma SAA, a marker of systemic inflammation, was measured by ELISA [14].

### Adipose Tissue – Stromal Vascular Fraction Separation and Flow Cytometry

Epididymal white adipose tissue (EWAT) from mice were excised at the time of sacrifice and minced in PBS. Minced samples were placed in HEPES-buffered DMEM supplemented with 10 mg/ml fatty acid–poor BSA and centrifuged at 1000 g for 10 minutes at room temperature to pellet erythrocytes and other blood cells. A lipopolysaccharide-depleted collagenase (type IV) cocktail (Sigma-Aldrich, St. Louis, MO) (1 mg/ml) was added to the tissue filtrate and the samples incubated at 37°C on an orbital shaker for 35 minutes. Once digestion was complete, samples were passed through a sterile 250-µm nylon mesh. The suspension was centrifuged at 500 g for 10 minutes. The pelleted stromal vascular cells (SVCs) were resuspended in erythrocyte lysis buffer and incubated at room temperature for 5 minutes. The erythrocyte-depleted SVCs were centrifuged at 500 g for 5 minutes. The SVC fraction was subject to FACS analysis, using antibodies that allow for differentiation between macrophage and T cell populations. For macrophages, the antibodies used were against F4/80, CD11b and CD11c, and cells were stained with propidium iodide (PI) to differentiate live cells. These cells were gated on live cells (those that were PI negative) and CD11b positivity. Subsequent determinations included the presence of F4/80 followed by the presence or absence of CD11c. The cells were analyzed on a BD FacsCANTO II flow cytometer utilizing FACS Diva Software. For T cells, the antibodies (purchased from eBiosciences, San Diego, CA and Biolegend, San Diego, CA) were against CD3, CD4, CD8, FoxP3, and CD62L. A commercially available FoxP3 buffer (eBiosciences, San Diego, CA) was utilized to allow intracellular staining for FoxP3. CD62L was used to differentiate activated T cells, as activation leads to shedding of CD62L. Hence, activated T cells were defined as CD62L negative. Single-cell suspensions of splenocytes and adipose tissue SVC were fixed & permeabilized (Foxp3 buffer set, eBioscience), then stained with fluorochrome-labeled antibodies against the above mentioned markers. Stained cells were analyzed on an LSR II flow cytometer (BD Biosciences) running FACSDiva software.

Relative proportions (% of total SVC cells) of activated CD4 or CD8 T cells (CD3^+^CD4/8^+^CD62L^−^) and regulatory T cells (CD3^+^CD4^+^Foxp3^+^) were enumerated by FACS analysis, and multiplied by the total number of SVC cells obtained per tissue to calculate the total number of that cell type per tissue. The number of cells was subsequently normalized to the amount of adipose tissue.

### Histological and Immunohistochemical Characterization of Tissues

Tissues were preserved in 10% formalin for 24 hours and fixed in paraffin. Sections were utilized for hematoxylin and eosin and Movat’s pentachrome staining as described previously [15]. Adipose tissues and livers were also stained with the macrophage-specific antibody Mac2. Immunohistochemical analysis was performed using techniques described previously [16].

### Real-time Quantitative Polymerase Chain Reaction of Adipose Tissue and Liver

Total RNA from harvested tissue (adipose tissue and liver) was extracted with a QIAGEN kit using standard protocols. After DNase treatment, 2 µg of RNA was reverse transcribed. cDNA generated was subjected to quantitative real time PCR amplification using the TaqMan Universal PCR Master Mix (Life Technologies, Grand Island, NY) reagent kit and ABI 7900 instrument, according to standard protocols. The primers and probes were obtained from Life Technologies. Co-amplification of mRNA for mouse *Gapdh* as the housekeeping gene was performed in all samples. Relative expression was assessed by the comparative C_T_ method.

### Glucose and Insulin Tolerance Testing

Glucose tolerance testing was performed on fasted mice by frequent sampling at 0, 30, 60 and 120 minutes by tail nicking after intra-peritoneal glucose injections at 13 weeks after initiating diets. The intraperitoneal glucose dose was 1 mg/kg. Mice were fasted for approximately 4 hours prior to testing. The insulin resistance index is the homeostatic model assessment of insulin resistance, calculated by multiplying the fasting glucose by the fasting insulin and dividing by 405, using mass units of mg/dl [17].

### Statistics

Data are expressed as means ± SEM unless noted otherwise. Mean values were compared using one-way ANOVA with Duncan’s post hoc testing on parametric data (with HFHSC as the control for interpretation purposes) using SPSS Statistics 20 (IBM, Somers, NY). Data not normally distributed were analyzed using the Kruskal-Wallis test with Dunn post-test using GraphPad Prism program (Version 5.04, GraphPad Software, Inc., San Diego, CA). p<0.05 was considered statistically significant.

## Results

### Weight Gain is Reduced in Mice Receiving Anti-CD40L Antibody

**Figure 1:**
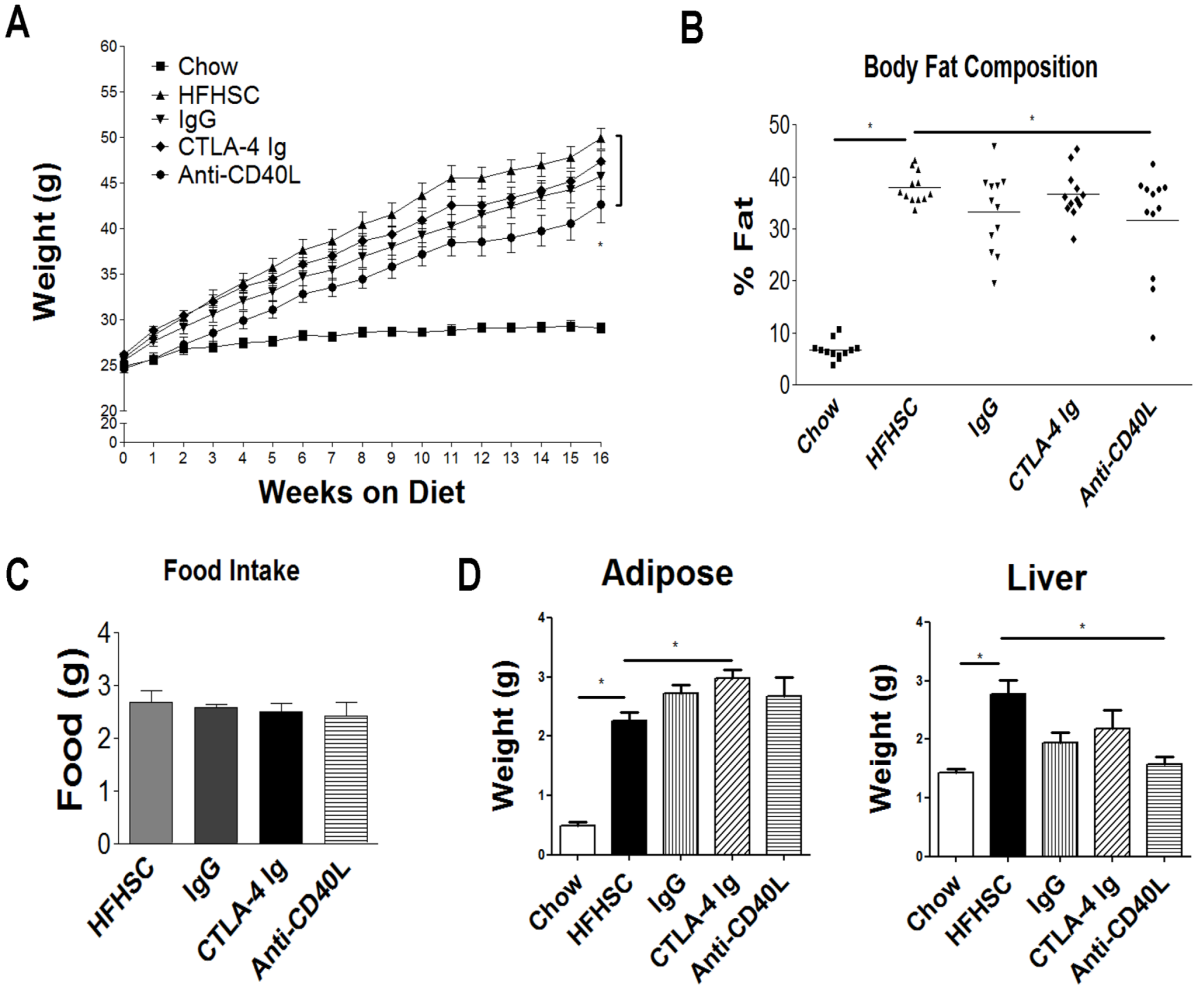
Body weight, body composition, food intake and organ weights. **A:** Weight data of mice over 16 weeks, Anti-CD40L Ab treated mice have significantly less weight gain compared to DDC controls. **B:** No differences between groups in body composition when measured at 6 weeks or 12 weeks. **C:** Food intake was comparable in all mice on the high fat diet. **D:** EWAT weights similar among mice on high fat diet, but liver weights are lower in mice receiving injections. n = 12 (* = p<.05).

Over a 16 week period, mice on the HFHSC diet gained a significant amount of weight compared to chow fed mice. This weight gain was similar among untreated mice on the HFHSC diet and mice treated with IgG and CTLA-4 Ig. Mice receiving the anti-CD40L antibody gained significantly less weight starting at the 10 week time point through the end of the study (Figure 1A). In addition to this difference in body weight, there was a significant difference noticed in the body fat composition of the mice treated with anti-CD40L antibody.(Figure 1B). Food intake also was similar among all mice on the HFHSC diet (Figure 1C). EWAT weights were similar among animals fed the obesogenic diet, but liver weights did differ in the treated mice as compared to the control high fat diet mice (Figure 1D). This difference in liver weights was not due to a difference in liver fat, as measured by liver triglycerides and oil red O staining (data not shown).

### Treatment with Anti-CD40L Antibody Lowers Serum Cholesterol

No change was observed in serum triglycerides among any of the animals. As expected, the cholesterol rose in the mice on the high fat, high cholesterol diet, and the co-stimulation blockade with anti-CD40L lowered cholesterol (Table 1). IgG increased circulating SAA levels in mice fed the high fat diets (Table 1), an effect not seen with T cell co-stimulation inhibitors.

**Table 1:** Serum Lipid and SAA Levels.

	Chow	HFHSC	IgG	CTLA-4 Ig	Anti-CD40L
Cholesterol (mg/dl)	73.28±2.44 (p<.05)	216.82±12.13	192.60±11.99	194.21±16.56	172.78±12.12 (p<.05)
Triglycerides (mg/dl)	43.33±2.14	44.04±3.48	44.46±4.04	50.47±4.13	45.72±6.73
SAA (µg/ml)	15.89±3.53	25.11±2.85	55.72±9.00 (p<.05)	31.00±5.55	25.93±3.18

The data are represented as mean ± SEM. n = 12.

### Co-stimulation Blockade Inhibits Obesity-associated CD8+ T Cell and Pro-inflammatory Macrophage Accumulation in Adipose Tissue

**Figure 2:**
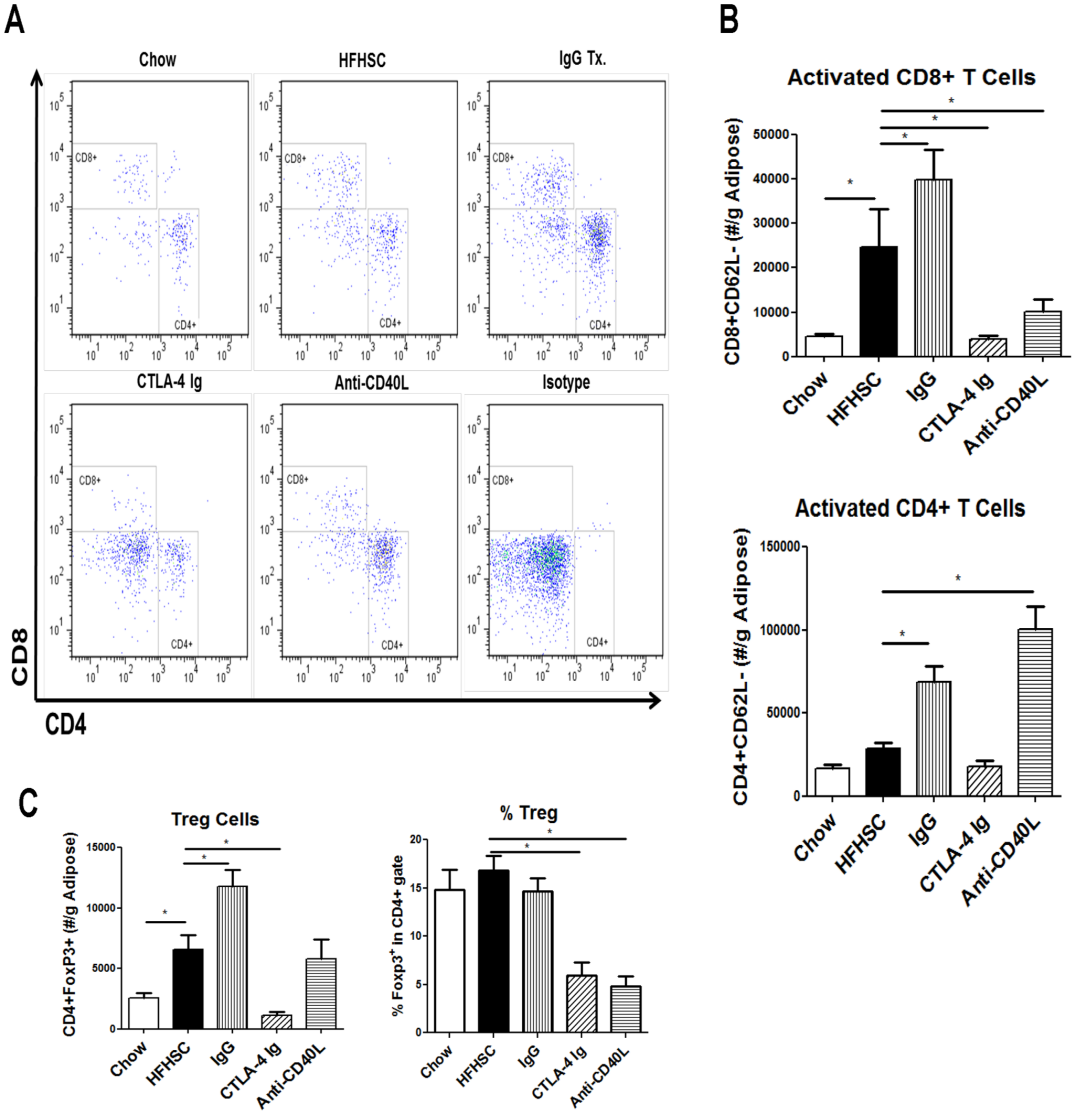
Adipose T Cell Flow Cytometry Analysis. **A:** Representative FACS plots of CD4 and CD8 T cell populations **B:** Graphical representation of activated CD8 and CD4 T cells. Activated T cells are those without CD62L. **C:** Treg cell populations. n = 9 (* = p<.05).

CD8+ T cells are typically pro-inflammatory. Consistent with previous reports, we also observed an increase of CD8+ T cells in adipose tissue of obese mice (Figures 2A and 2B). Treatment with both CTLA-4 Ig and anti-CD40L antibody reduced their numbers (Figure 2B). We performed analysis using different forms of normalization and did not see a difference in interpretation. We provide the figures showing number of cells per gram of adipose tissue.

Activated CD4+ T (CD4+CD62L-) cells were also decreased in mice treated with CTLA-4 Ig, but increased in mice receiving anti-CD40L (Figure 2B). We did not discern between pro-inflammatory Th1 CD4+ T cells and anti-inflammatory Th2 CD4+ T cells. However, we did stain for Foxp3, a marker of anti-inflammatory CD4+ T regulatory cells. Despite previous studies that indicated T regulatory cells were enriched in lean adipose tissue versus obese adipose tissue [9], we showed an increased number of T regulatory cells in obese adipose tissue, and the total number declined when mice were treated with CTLA-4 Ig and anti-CD40L (Figure 2C). However, when expressed as a percentage of cells in the CD4+ gate, there was no difference in T regulatory cells between the chow and HFHSC fed mice, yet there was a significant decline in the mice treated with CTLA-4 Ig and anti-CD40L (Figure 2C). Representative FACS plots of T regulatory cells in adipose tissue are shown in Figure S1.

**Figure 3:**
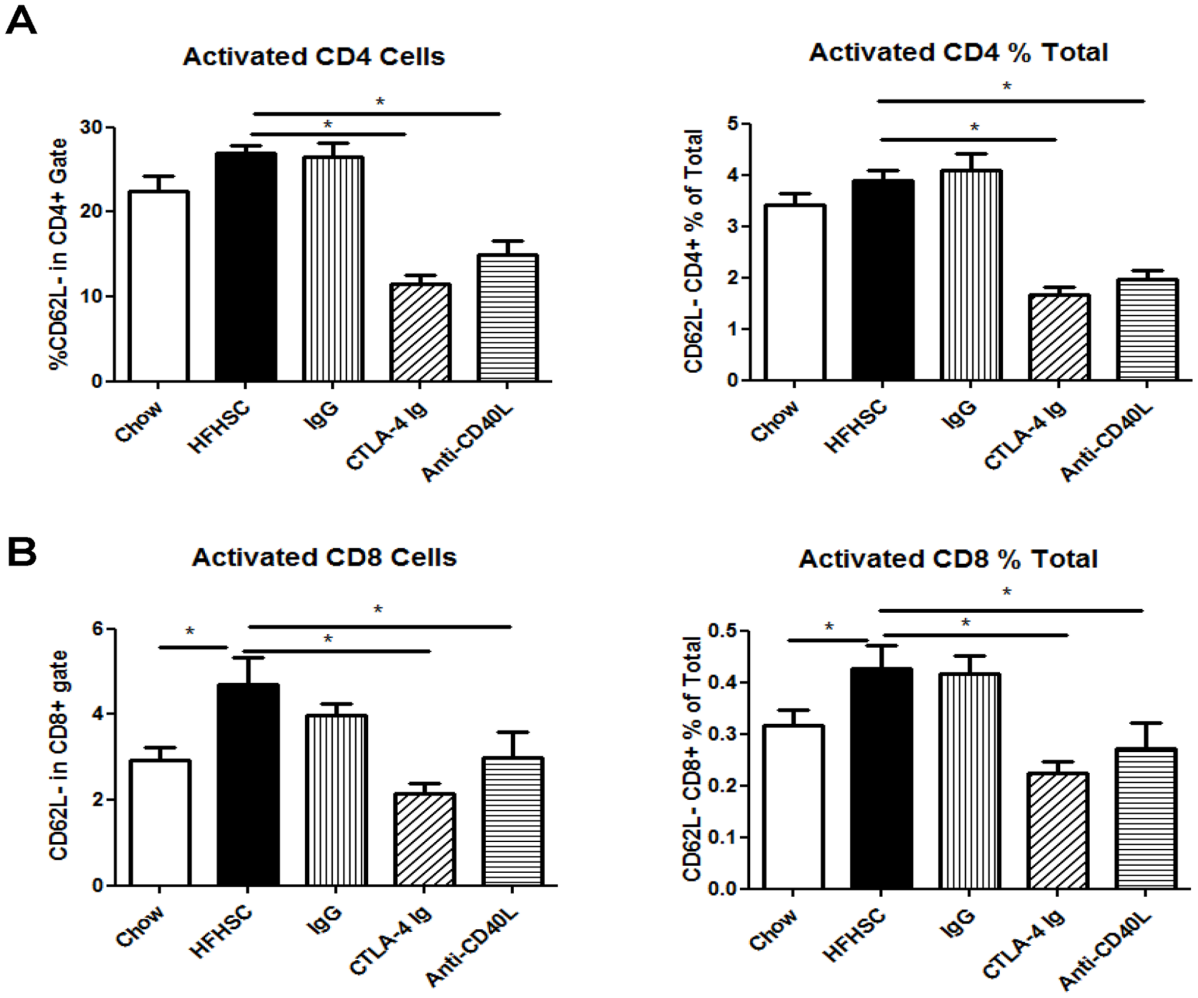
Spleen T Cell Flow Cytometry Analysis. **A:** Activated CD4 T cells in the spleens of mice (n = 9), represented as a percentage of cells in the CD4 gate and as a percentage of total cells. **B:** Activated CD8 T cells in the spleens of mice (n = 9), represented as a percentage of cells in the CD8 gate and as a percentage of total cells. (* = p<.05).

In addition to evaluating the T cells in adipose tissue, we performed flow cytometry to assess T cells in the spleen. This gave an indication of systemic T cell activation. In accordance with expectations, the mice treated with CTLA-4 Ig and anti-CD40L had reduced peripheral T cell activation in both CD4+ and CD8+ populations (Figure 3A and 3B). This provides evidence that the treatments were performing as anticipated.

**Figure 4:**
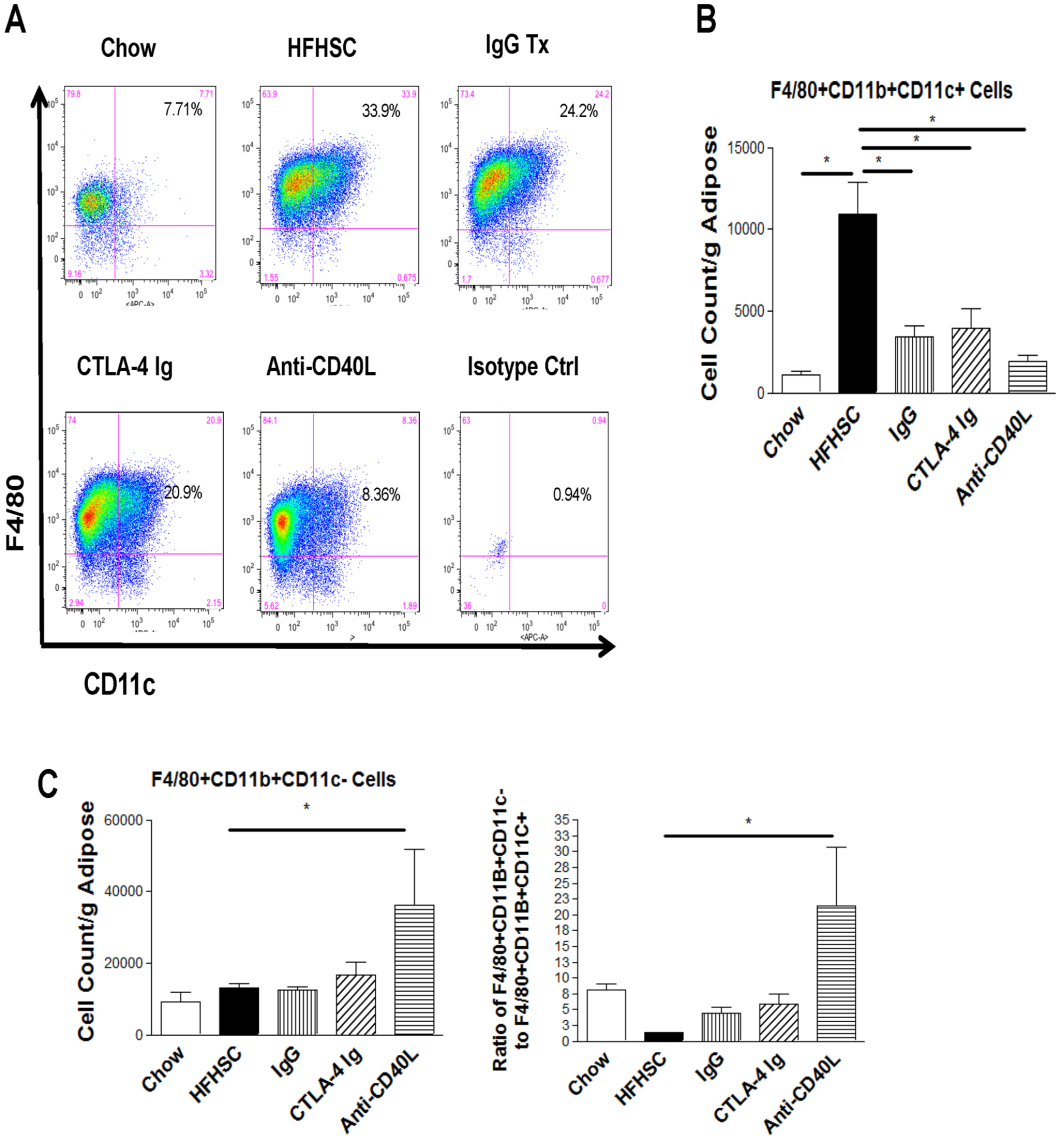
Adipose Macrophage Flow Cytometry Analysis. **A:** Representative FACS plots of pro-inflammatory macrophage, designated as triple positive cells, staining positive for F4/80, CD11b and CD11c. These cells are already gated on CD11b positivity. Please see methods for description of gating strategy. **B:** Graphical representation of pro-inflammatory macrophage. C: Graphical representation of anti-inflammatory macrophage and ratio of anti-inflammatory to pro-inflammatory cells. n = 6 (* = p<.05).

Mice that received injections had less F4/80+CD11b+CD11c+ macrophages, which are considered to be pro-inflammatory (Figures 4A and 4B). Mice treated with anti-CD40L antibody also had much more F4/80+CD11b+CD11c- cells, which are consistent with anti-inflammatory macrophages (Figure 4C). In addition, the ratio of anti-inflammatory to pro-inflammatory macrophages are improved significantly in anti-CD40L antibody treated mice and have an upward trend in the mice receiving IgG and CTLA-4 Ig (Figure 4C). Interestingly, mice treated with IgG actually had higher levels of CD8+ T cells than those treated with CTLA-4 Ig and anti-CD40L antibody, yet also exhibited reduced pro-inflammatory macrophages. This suggests a separate mechanism of action for IgG in reducing the pro-inflammatory macrophage numbers.

### Immunohistochemistry of Adipose Tissue Depicts Decreased Adipose Tissue Macrophage Content in Injected Mice

**Figure 5:**
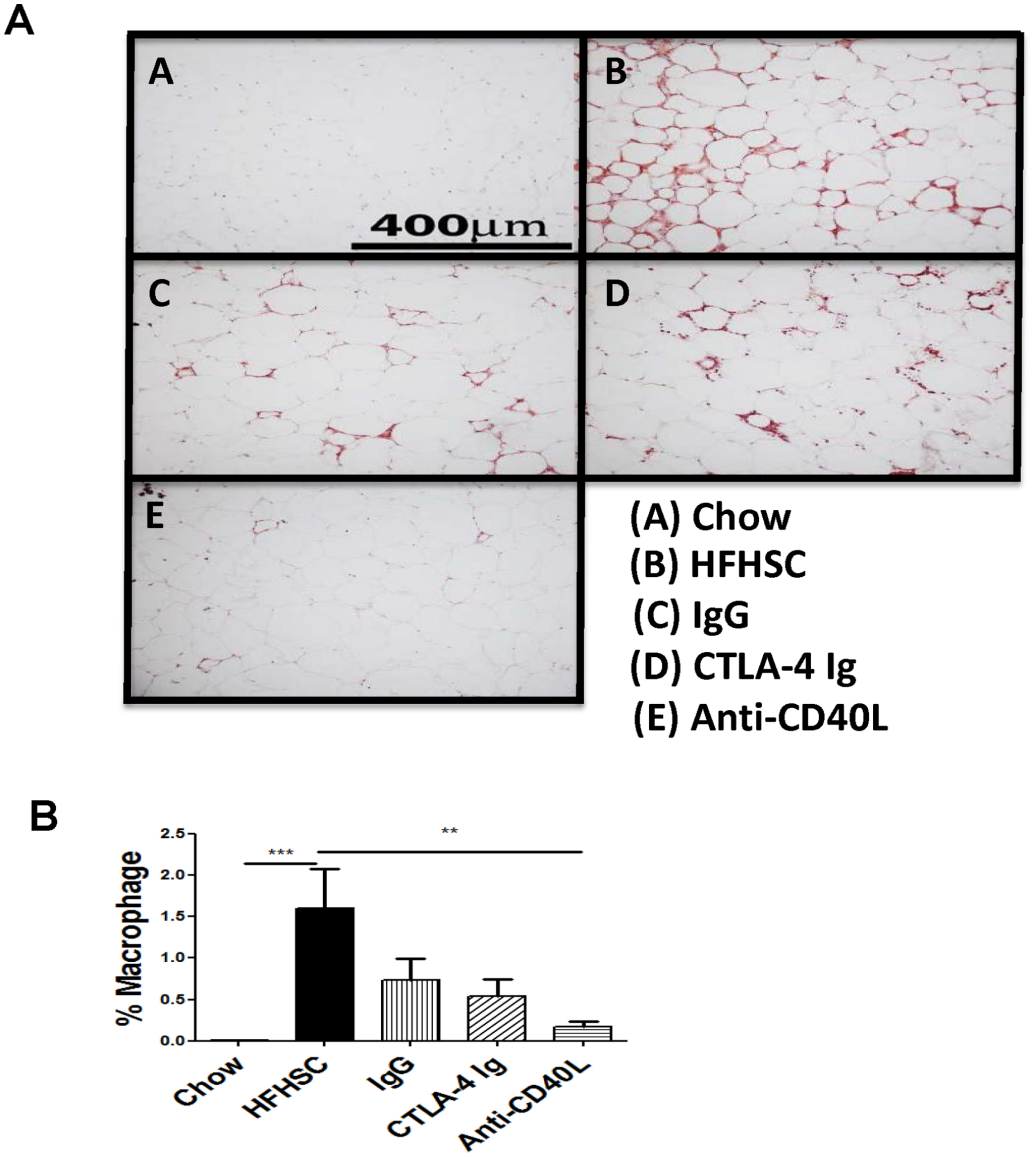
Immunohistochemistry of Adipose Tissue. The epididymal adipose tissue was stained for Mac2 depicts decreased numbers of macrophages in treated mice. **A:** Representative photomicrographs of the tissue stains. **B:** Graphical representation of macrophage numbers in each group of mice. n = 12 (* = p<.05, ** = p<.01, and *** = p<.001).

EWAT was stained for Mac2, a pan macrophage marker. The photomicrographs in Figure 5A show decreased macrophage staining in the treated mice. The graphical representation in Figure 5B identifies decreased macrophage numbers by statistical analysis, with significant findings in mice treated with anti-CD40L, and a trend toward decrease in the other treated mice. These results are consistent with the flow cytometry findings.

### Gene Expression Analysis Indicates Reduced Pro-inflammatory Macrophages in Adipose Tissue of Mice Treated with T Cell Co-stimulatory Inhibitors

**Figure 6:**
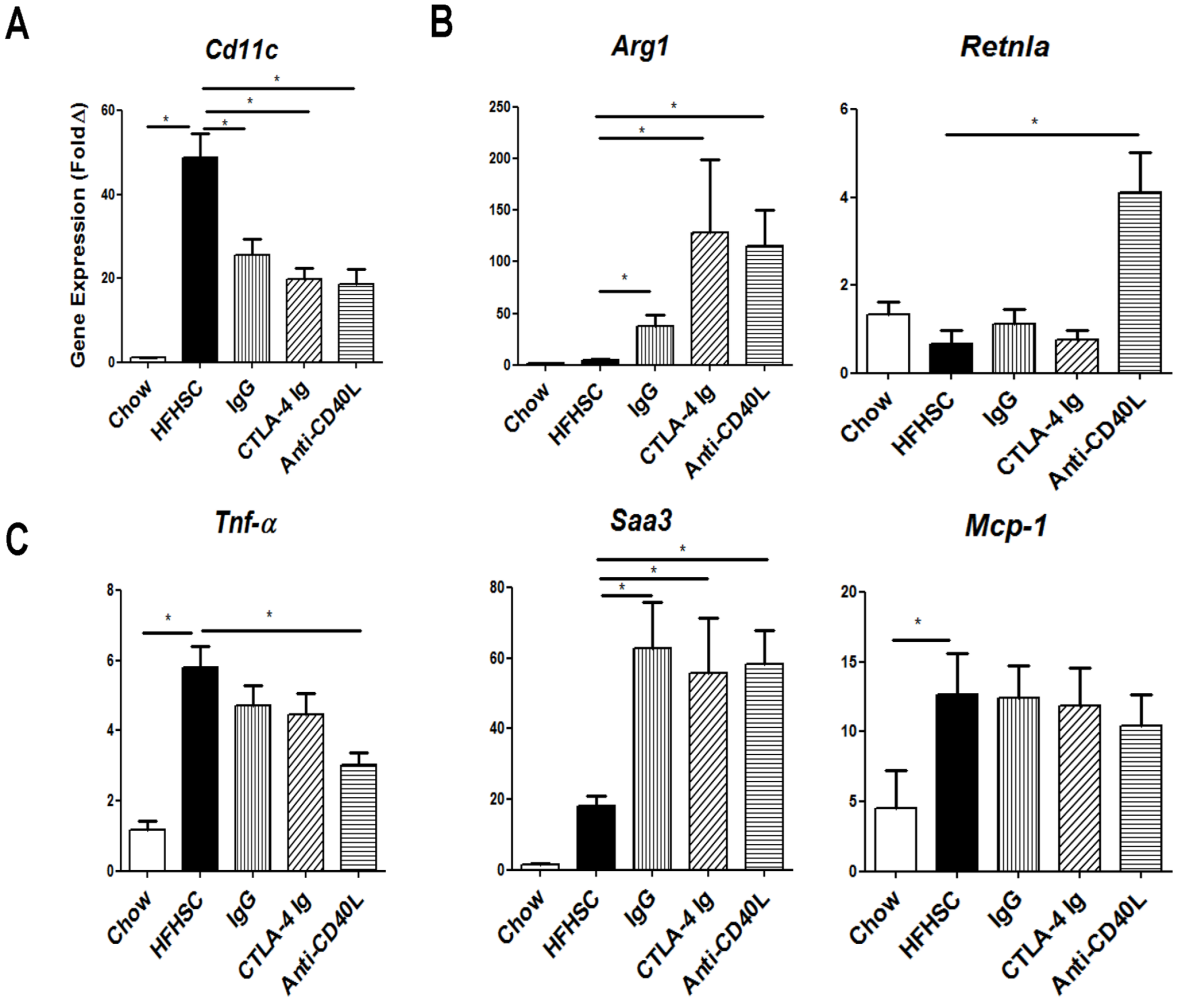
Adipose Gene Expression. **A:** Adipose gene expression corroborates FACS findings of decreased pro-inflammatory macrophages in treated mice, as *Cd11c* is decreased. **B:** Anti-inflammatory markers, *Arg1* and *Retnla*, are elevated in treated mice. **C:** Also consistent with decreased pro-inflammatory macrophages, *Tnf-α* is decreased. However, *Saa3* is markedly elevated in treated mice, and no difference is observed in *Mcp-1* among treated mice. n = 11–12 (* = p<.05).

This analysis corroborated our FACS findings, in that one of the markers for pro-inflammatory macrophages, *Cd11c*, had reduced gene expression in treated mice (Figure 6A). The anti-inflammatory marker, *Arg1*, was elevated in all of the treated mice, compared to the HFHSC control (Figure 6B). Another anti-inflammatory marker, *Retnla*, was significantly elevated only in the mice treated with anti-CD40L (Figure 6B). The gene expression analysis showed that diet-induced adipose tissue expression of the pro-inflammatory cytokine, *Tnf-α,* was attenuated in treated mice (Figure 6C). Of striking interest, a separate marker of inflammation, *Saa3*, exhibited a marked increase in adipose tissue gene expression in treated mice (Figure 6C). We were unable to detect any significant differences among treated mice in other pro-inflammatory cytokines such as *Mcp-1* (shown in Figure 6C), *Il-6* or *Ifn-γ* (data not shown). The issue with detecting gene expression changes in *Il-6* and *Ifn-γ* is due to dilution of these genes in whole adipose tissue which was used for this analysis. Use of SVC cells would have been preferable for this analysis, but they were used for the flow cytometry analysis.

**Figure 7:**
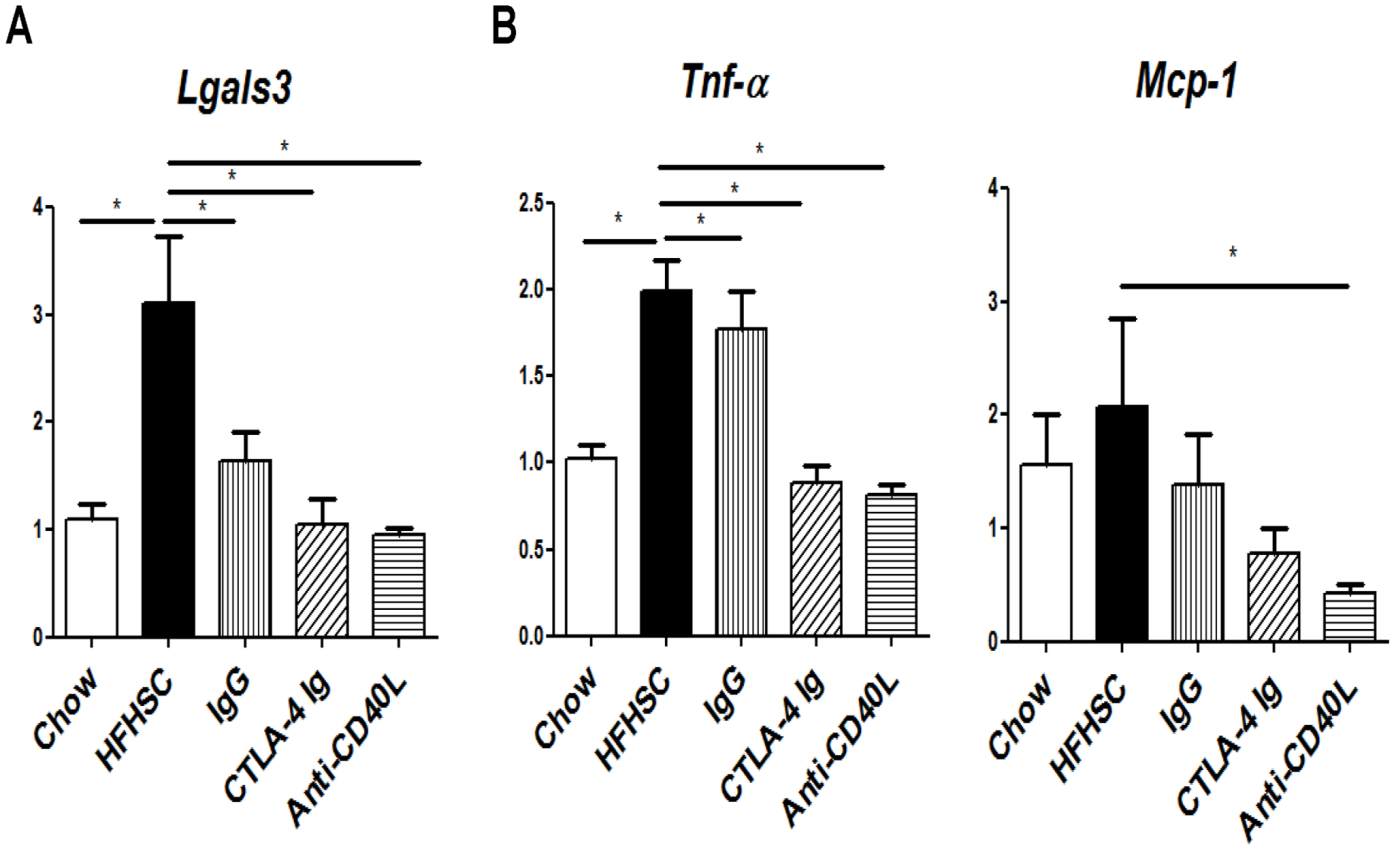
Liver Gene Expression. **A:** Macrophage marker, *Lgals3*, is decreased in liver of all treated mice. **B:** Expression of pro-inflammatory cytokine, *Tnf-α* is decreased in mice treated with CTLA-4 Ig and anti-CD40L. *Mcp-1* is decreased in mice treated with anti-CD40L, and trending toward decreased in the other two treatment groups. N = 11–12 (* = p<.05).

Liver gene expression analysis showed reduced *Lgals3, Tnf-α* and *Mcp-1* expression, suggesting a decrease in inflammation in the liver as well as in adipose tissue (Figures 7A and 7B).

### Inhibition of T Cell Co-stimulation did not Affect Glucose Tolerance

**Figure 8:**
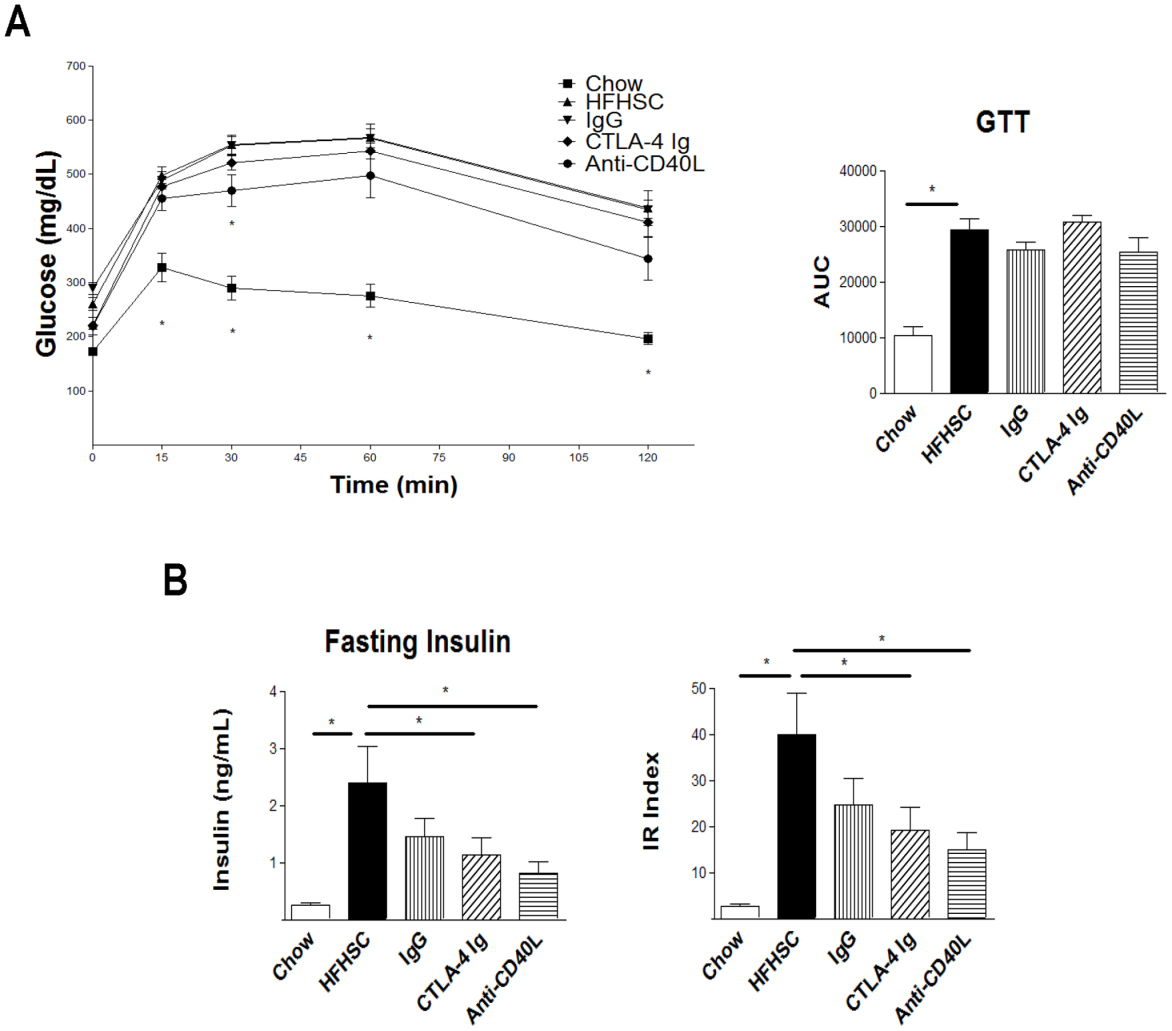
Metabolic Parameters. **A:** Glucose tolerance not improved as measured by GTT area under the curve (completed during week 13 of the study, n = 12). There was a trend toward improvement in the mice treated with anti-CD40L antibody. **B:** By the end of the study, the mice treated with anti-CD40L antibody have lower fasting insulin and insulin resistance (IR) index. The IR index is a measure of insulin resistance based on a ratio of fasting insulin and glucose, which is essentially the homeostatic model assessment of insulin resistance. n = 12 (* = p<.05).

Glucose tolerance was evaluated with an intraperitoneal GTT. The mice treated with anti-CD40L antibody had a trend toward improvement, but the AUC was not statistically significant (Figure 8A). Despite the lack of improvement in glucose tolerance, we did observe an improvement in fasting insulin and insulin resistance index in the mice treated with anti-CD40L antibody at the 16 week time point (Figure 8B).

## Discussion

**Figure 9:**
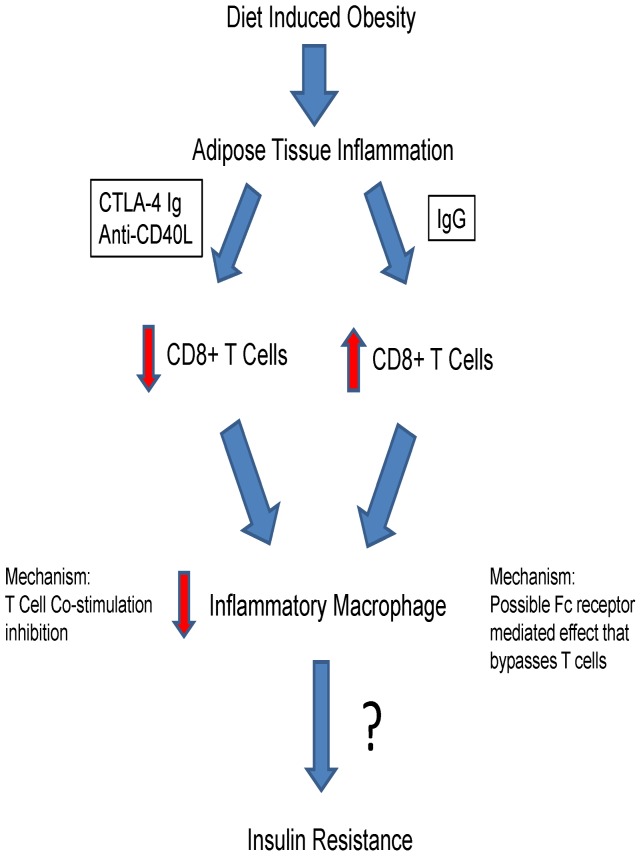
Hypothetical Model by which inhibition of T cell co-stimulation affects adipose tissue inflammation. Anti-CD40L antibody and CTLA-4 Ig reduce activated CD8+ T cells in adipose tissue, working as expected to reduce T cell co-stimulation/activation. The importance of CD8+ T cells in adipose tissue inflammation has been previously explained [7]. IgG treatment inexplicably increased CD8+ T cells. Despite the differential effects on CD8+ T cells, the pro-inflammatory macrophage content in adipose tissue decreased with all three treatments. This suggests that the IgG effect is due to a different mechanism of action than any potential unknown effect on T cells. Furthermore, despite this presumably beneficial effect on macrophages, an effect on insulin resistance remains questionable.

We have shown that adipose tissue inflammation can be reduced by administration of co-stimulatory inhibitors. The most remarkable finding was the reduction in the activation of one of the most potent pro-inflammatory T cell populations, the CD8+ T cell population. This occurred with both CTLA-4 Ig and anti-CD40L, whereas the injection of IgG appeared to increase this population of T cells. In conjunction with these results, we saw a reduction in pro-inflammatory macrophages, designated as F4/80+CD11b+CD11c+ cells. A hypothetical model to depict how inhibition of T cell co-stimulation affects adipose tissue inflammation is shown in Figure 9. One of the most common macrophage and adipocyte cytokine genes elevated in inflamed adipose tissue is *Tnf-α*. Consistent with the reduction in macrophages, gene expression analysis revealed reduced expression of *Tnf-α* in adipose tissue after co-stimulation blockade. In addition, *Tnf-α* and *Lgals3* gene expression were also reduced in liver, suggesting the inflammatory response is also being modulated in that tissue as well, which would be expected, as the molecules were injected systemically. Overall, the results indicate that systemic administration of co-stimulation inhibitors alters adipose and liver inflammation, confirming part of the original hypothesis.

One of the agents, anti-CD40L, also induced a decreased weight gain and body fat composition compared to the HFHSC animals that did not receive an injection. Food intake was equivalent, which suggests higher energy expenditure or reduced nutrient absorption. This is similar to published data using a CD40L knockout mouse model, as well as the same clone of anti-CD40L antibody [18]. Future studies with anti-CD40L antibody in obesity will need to evaluate its effect on energy expenditure. As previously mentioned, the ligation of CD40 augments the antigen presenting function of multiple APC such as macrophages, dendritic cells and B cells. With regards to B cells, CD40 ligation also allows for isotype switching of antibodies, giving the B cell the ability to produce an effective antibody response. In addition, there are other sources of CD40L, such as platelets, and other cell types that express CD40, such as adipocytes. Given the differential findings among the treatments used, it is very likely that blocking the CD40-CD40L pathway has effects on multiple facets of inflammation signaling beyond T cell co-stimulation. Furthermore, since CD40-CD40L deficiency can result in immunodeficiency in humans [19], blocking this pathway might have resulted in adverse events such as an undefined infection that could result in weight loss or lack of weight gain. Although a consideration, it is less likely due to the extensive efforts maintained to minimize infections in the vivarium.

Interestingly, the reduction of T cell activation was associated with a reduction in pro-inflammatory macrophages. The one caveat to that was the reduction in macrophages seen in mice given the IgG protein, where they actually had an increase in activated CD8+ T cells. We believe the reduced pro-inflammatory macrophage count was due to a separate mechanism. Intravenous immunoglobulin (IVIG) is a therapy used for autoimmune disorders, where overwhelming the system with non-pathogenic antibody appears to elicit a beneficial response, an effect that may be Fc receptor mediated [20]. In our study, we believe that the Fc receptors on macrophage are being affected by the Fc region on the non-pathogenic IgG protein. This is not an issue for CTLA-4 Ig, since the Fc region of the molecule is mutated and cannot bind the Fc receptors in the mouse. The reason this is not an issue for the anti-CD40L antibody, MR1, is that the antibody does not activate the mouse Fc receptor [21]. So these observations are unique to the IgG protein, whereas the observations with the other proteins are likely due to the reductions in T cell activation. This effect of the control IgG protein is speculative since the use of IVIG involves high doses of protein that contain multiple IgG isotypes.

A limitation of our study was the lack of a saline injection control, as we expected the IgG to be our control. However, we did have the untreated mice for comparison. Another limitation is that we did not differentiate between Th1 and Th2 CD4+ T cells, since differences in CD4+ T cell activation were observed between our two co-stimulatory blockers. In the CTLA-4 Ig group, we saw decreased CD4+ T cell activation, which could be due to a reduction of either Th1 and/or Th2 cells. In the anti-CD40L group, we saw an increase in the activated CD4+ T cells. Since the treatment was seemingly beneficial, we can presume this represents an increase in the anti-inflammatory Th2 cells.

An additional interesting finding was the effect of anti-CD40L antibody on cholesterol levels in mice fed the obesogenic diet, the reason for which is not immediately apparent. There appears to be a link between inflammation and cholesterol metabolism seen in other studies of mice lacking TLR4 and CD180, two molecules that are involved with propagation of inflammatory signals [22], [23]. Blocking inflammation by various mechanisms may interfere with cholesterol metabolic pathways in a yet undiscovered manner. This is an area that needs further exploration.

Other interesting findings in this study include the increased FoxP3+ CD4+ T regulatory cells in adipose tissue from untreated obese mice versus lean chow fed mice, which were subsequently decreased further in the CTLA-4 Ig group and unchanged in the anti-CD40L group. This contradicts previous reports that suggest T regulatory cells decrease in obesity [9]. Since T regulatory cells are anti-inflammatory, it would make theoretical sense that they try to dampen the inflammatory response during obesity, but may not be able to overcome that challenge. CTLA-4 Ig is known to reduce T regulatory cell activation when used alone [24], which might explain the finding in this study. However, the use of CTLA-4 Ig in combination with anti-CD40L can result in an induction of T regulatory cells. This has been reported in an acute inflammatory setting with a strong antigenic stimulus [25]. This is an area that needs further study in the presence of a chronic low grade inflammation, such as is seen with obesity.

The lack of major improvement in glucose tolerance was unexpected given the decreased pro-inflammatory macrophage and TNF-α gene expression, and because a previous report evaluating CD40L deficiency did find an improved glucose tolerance in the mice treated with anti-CD40L [18]. A potential extraneous variable is the diet used in this study, which is very inflammatory due to the added cholesterol and large amount of sucrose. This has the potential to confound the metabolic results [26]. An alternative interpretation of our data is that adipose tissue inflammation does not contribute directly to whole body insulin resistance, even though there may be compensatory inflammatory responses occurring in adipose tissue that are associated with insulin resistance. In addition, since others have concluded that T regulatory cells are involved in adipose tissue inflammation and insulin resistance [9], [27], [28], the fact that the treatments used here reduce these cells may have been detrimental to a potential improvement in insulin resistance despite the other perceived benefits of the treatments. Another important factor is the number of injections the mice received throughout the course of the study. This may have led to a high amount of stress that may confound glucose metabolism results that are closely linked to stress levels, particularly in mice.

Obesity-associated adipose tissue inflammation is characterized by the accumulation of multiple inflammatory cells in adipose tissue, not just T cells and macrophages. Although we found beneficial responses of T cells and macrophages with T-cell co-stimulation inhibition, we did not evaluate all of the possible permutations of immune responses, which was beyond the scope of the present study. However, we observed an interesting finding related to SAA3, which represents a marker for inflammation. SAA3 is an extra-hepatic form of SAA, which is expressed in both adipocytes and macrophages [29], [30], and is increased in whole adipose tissue in diet-induced obesity [31]. However, despite the improvement in adipose tissue inflammation in our study, *Saa3* gene expression actually increased markedly in animals treated with both T-cell co-stimulation inhibitors. This finding was unexpected, and may signify a role for a compensatory inflammatory response. Alternatively, since this was seen in all mice receiving injections, we cannot rule out that this was a function of the injections themselves, either physically or from a contamination standpoint. The main consideration for contamination would be lipopolysaccharide. However, if that were the case, we would expect *Tnf-α* gene expression to rise in parallel, whereas we observed the opposite effect. Since the arsenal of the immune system is vast, it would appear that a multi-pronged approach would be necessary to interfere with the inflammation occurring in obesity. However, a multi-pronged approach would result in multiple modes of immune suppression. The potential benefits of this suppression would need to be weighed against the risks to the host defense mechanism.

Atherosclerosis is another co-morbidity associated with obesity and insulin resistance. There are parallels between the inflammation seen in adipose tissue and the artery wall. Both obesity and atherosclerosis are chronic inflammatory diseases propagated by components of both the innate and adaptive immune systems [32], [33]. Previous examination has revealed the presence of activated T cells in human atherosclerotic plaques [34]. Since we observed a reduction in CD8+ T cells and macrophages in adipose tissue after co-stimulation blockade, it is possible that the same may occur in artery wall. Since lipid parameters were unchanged, this would be expected to impart a benefit only in the inflammatory arm of atherosclerosis. Therefore, it would be important to determine the degree of potential benefit that these therapies could induce in atherosclerosis. Anti-CD40L has previously been shown to improve atherosclerosis [35]. To our knowledge, CTLA-4 Ig has not been evaluated in this setting, nor has the combination of both immunotherapies, which would be of interest to evaluate in an atherosclerotic mouse model. These types of studies can provide not only a proof of concept, but can provide practical implications for therapy as well. As previously discussed, CTLA-4 Ig is already an approved clinical therapy for the treatment of rheumatoid arthritis. Drugs that target CD40L are being developed for use in human autoimmune disease, which will likely become reality in the near future. Studying these molecules may pave the way for future therapeutic use to combat the inflammatory responses seen in obesity and atherosclerosis.

## Supporting Information

Figure S1 **Representative FACS plots of T regulatory cells.** CD4 and Foxp3 staining on gated CD3+ cells. As expected, the Foxp3 antibody stains a sub-population of CD4+ T cells, but none of the CD4^neg^ (i.e. CD8+) T cells. This plot is provided to complement the data shown in Figure 2C.
(TIFF)
