# Supplementary figures and images for: T Cell Activation Inhibitors Reduce CD8+ T Cell and Pro-Inflammatory Macrophage Accumulation in Adipose Tissue of Obese Mice
Source: PLoS One. 2013 Jul 2;8(7):e67709. doi: 10.1371/journal.pone.0067709 (PMC3699637; doi:10.1371/journal.pone.0067709)

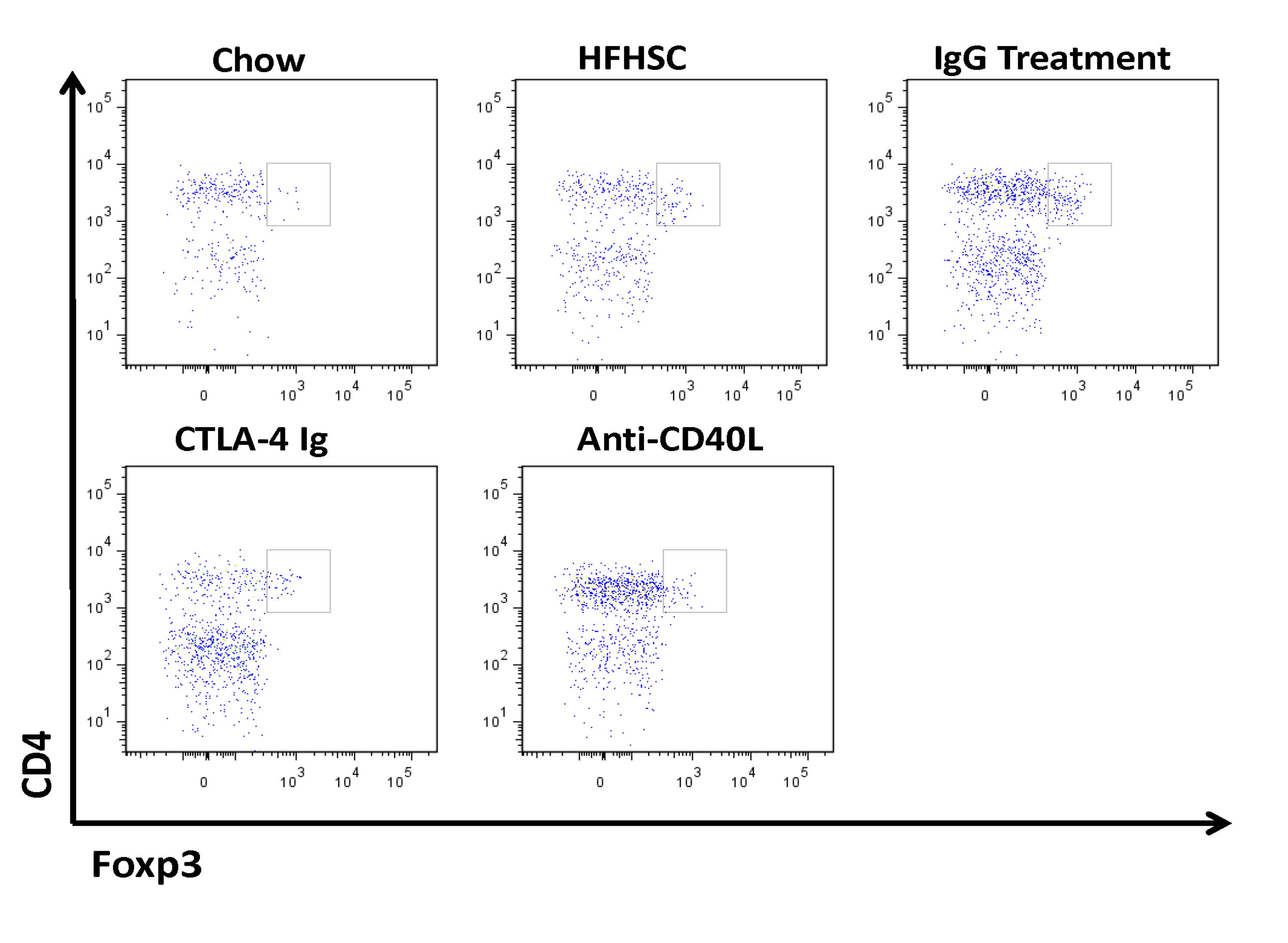

Supplement: Figure S1 — Representative FACS plots of T regulatory cells. CD4 and Foxp3 staining on gated CD3+ cells. As expected, the Foxp3 antibody stains a sub-population of CD4+ T cells, but none of the CD4neg (i.e. CD8+) T cells. This plot is provided to complement the data shown in Figure 2C. (TIFF) [file pone.0067709.s001.tiff]
